# *Waxy* and *non-waxy* barley cultivars exhibit differences in the targeting and catalytic activity of GBSS1a

**DOI:** 10.1093/jxb/erw503

**Published:** 2017-02-11

**Authors:** Kim H. Hebelstrup, Morten Munch Nielsen, Massimiliano Carciofi, Olga Andrzejczak, Shahnoor Sultana Shaik, Andreas Blennow, Monica M. Palcic

**Affiliations:** 1 Department of Molecular Biology and Genetics, Section of Crop Genetics and Biotechnology, Aarhus University, Forsøgsvej 1, 4200 Slagelse, Denmark; 2 Carlsberg Laboratory, Gamle Carlsberg Vej 10, DK-1799 København V, Denmark; 3 Department of Plant and Environmental Sciences, Faculty of Science, University of Copenhagen, Thorvaldsensvej 40, 1871 Frederiksberg C, Denmark

**Keywords:** Amylose, GBSS, starch biosynthesis, starch functionality, subcellular targeting, waxy.

## Abstract

Amylose synthesis is strictly associated with activity of granule-bound starch synthase (GBSS) enzymes. Among several crops there are cultivars containing starch types with either little or no amylose known as *near*-*waxy* or *waxy*. This (near) amylose-free phenotype is associated with a single locus (*waxy*) which has been mapped to GBSS-type genes in different crops. Most *waxy* varieties are a result of either low or no expression of a GBSS gene. However, there are some *waxy* cultivars where the GBSS enzymes are expressed normally. For these types, single nucleotide polymorphisms have been hypothesized to represent amino-acid substitutions leading to loss of catalytic activity. We here confirm that the HvGBSSIa enzyme from one such *waxy* barley variety, CDC_Alamo, has a 90% reduction in catalytic activity. We also engineered plants with expression of transgenic C-terminal green fluorescent protein-tagged HvGBSSIa of both the *non-waxy* type and of the CDC_Alamo type to monitor their subcellular localization patterns in grain endosperm. HvGBSSIa from *non-waxy* cultivars was found to localize in discrete concentric spheres strictly within starch granules. In contrast, HvGBSSIa from *waxy* CDC_Alamo showed deficient starch targeting mostly into unknown subcellular bodies of 0.5–3 µm in size, indicating that the waxy phenotype of CDC_Alamo is associated with deficient targeting of HvGBSSIa into starch granules.

## Highlight

The waxy phenotype of barley CDC_Alamo is associated with a reduction of catalytic activity and deficient targeting of GBSS1a.

## Introduction

Starch is the main polysaccharide in which carbon and energy are stored in higher plants and is the principal dietary source of energy for humans (Zeeman *et al.*, 2010; Tetlow, 2011). Cereal starch accounts for over 90% of the world market for starch (Tetlow, 2011) and is used in several industrial processes including papermaking and first generation bioethanol. Starch is an insoluble, semi-crystalline glucan composed of two polymers of glucose—amylose and amylopectin—that are linked together by α-1,4-D-glycosidic linkages with branch points formed by α-1,6-D-glycosidic linkages. The highly branched amylopectin is the major component of starch and has an estimated molecular mass of 10^7^–10^9^ Da whereas the essentially linear amylose is smaller than amylopectin (10^5^–10^6^ Da) (Buléon *et al.*, 1998). The ratio between amylose and amylopectin in plant starches varies depending on the botanical source, but typically 15–30% of the starch granule is composed of amylose (Tetlow, 2011). This ratio seems to have been optimized in nature for optimal starch granule robustness, digestibility and carbohydrate remobilization as demonstrated for barley seedling establishment (Shaik *et al.*, 2014). *Waxy* starches are characterized by either being amylose-free (*waxy*) or having low amylose content (*near-waxy*) (<5%) (Domon *et al.*, 2002). The texture of cooked starches is influenced by the content of amylose, and the clear starch pastes with good freeze–thaw stability and little tendency to retrograde that are formed by *waxy* starches are of high value in many processed foods (Denyer *et al.*, 2001). Also, the cloning and characterization of starch modifying enzymes is increasingly important for industrial and agro-biotechnological modification of starches and other carbohydrates (Hebelstrup *et al.*, 2015).

Starch synthesis in cereals involves five classes of enzymes: ADP-glucose pyrophosphorylase (AGPase), starch synthases (ADP-α-D-glucose:[1→4]-α-D-glucan 4-α-D-glucosyltransferase; EC 2.4.1.21, GT5), starch phosphorylase, and starch branching and debranching enzymes (Blennow *et al.*, 2013). Five classes of starch synthases have been identified in barley: granule-bound starch synthase (GBSSI) and starch synthases SSI, SSII, SSIII, and SSIV). All five classes catalyse the transfer of a glucosyl unit from ADP-glucose to the non-reducing end of an existing α-glucan chain (Zeeman *et al.*, 2010). Pioneering work in the 1960s suggested that amylose synthesis is strictly associated with activity of granule-bound starch synthase(s) (reviewed by Nelson and Pan, 1995). Mutations in *GBSSIa* genes have later been verified in many *waxy* mutants of cereal crops, where in particular *waxy* corn (Wessler and Varagona, 1985; Nelson and Pan, 1995) and glutinous rice (Wang *et al.*, 1995; Olsen and Purugganan, 2002) are the major *waxy* crops produced. Of the two GBSS genes in barley, only HvGBSSIa is expressed in the endosperm (Radchuk *et al.*, 2009), whereas HvGBSSIb is expressed in the pericarp and the embryo (Sreenivasulu *et al.*, 2006). GBSS-type enzymes represent the majority of the protein fraction bound within storage starch granules (Wang *et al.*, 2013), but they do not contain known types of starch binding domains. HvGBSSIa is targeted to amyloplasts by the recognition of an N-terminal transit peptide, which is cleaved off during translocation across plastid membranes. We have previously shown that the first 69 N-terminal amino acids of HvGBSSIa corresponds to such a transit peptide, which can effectively target transgenic enhanced green fluorescent protein (eGFP) to the amyloplast of barley endosperm cells (Hebelstrup *et al.*, 2010). In Arabidopsis, targeting of GBSS to transient leaf starch granules has been shown to be dependent on interaction with PTST (protein targeting to starch), which is a 26 kDa protein containing a glucan-binding family 48 carbohydrate-binding module (CBM48) (Seung *et al.*, 2015). In barley, storage starch accumulates in the starchy endosperm starting around 6 days after pollination and continues throughout the grain-filling period (Radchuk *et al.*, 2009). During grain development the amylose content increases from around 20% to around 30% in the mature grain (Radchuk *et al.*, 2009). The loss of activity from GBBSIa in barley was linked to the waxy phenotype by Hylton *et al.* (1996) and several *waxy* barley cultivars have since then been identified and characterized (Domon *et al.*, 2002; Patron *et al.*, 2002; Ma *et al.*, 2010; Asare *et al.*, 2012). Most *waxy* barley mutants are a result of either low or no expression of the *GBSSIa* gene caused by deletions or non-silent single nucleotide polymorphisms (SNPs) (Domon *et al.*, 2002; Patron *et al.*, 2002). However, there are some *waxy* cultivars with no detectable amylose wherein the full *GBSSIa* gene is expressed at a normal level, and therefore non-silent SNPs in the *GBSSIa* gene are interpreted to result in catalytic inactivity without affecting enzyme concentration (Asare *et al.*, 2012). However, the enzymes of these presumed loss-of-function alleles were never cloned and characterized *in vitro*. CDC Alamo is an example of such a *waxy* barley cultivar, where HvGBSSIa is expressed at a normal level (Patron *et al.*, 2002; Asare *et al.*, 2012). Here we describe what effect the SNPs identified in the *HvGBSSIa* gene of the *waxy* barley cultivar CDC Alamo have on the catalytic properties of HvGBSSIa and its subcellular localization. We found that HvGBSSIa from *non-waxy* cultivars are effectively targeted into concentric spheres within developing starch granules. In contrast, HvGBSSIa from the *waxy* cultivar CDC Alamo has a 90% loss of catalytic activity, and it is not correctly localized in starch granules. Instead it is localized mostly into unknown subcellular bodies with a size of 0.5–3 µm.

## Material and methods

### Constructs for expression of barley granule-bound starch synthases Ia

Barley granule-bound starch synthase (HvGBSSIa, GenBank accession: AAM74048) was codon optimized for expression in *E. coli* and synthesized by DNA2.0 (www.DNA20.com). It was synthesized without the initial 69 amino acid residues, i.e. plastid transit peptide as predicted using TargetP (http://www.cbs.dtu.dk/services/TargetP/). The *HvGBSSIa* gene contained additional *Nde*I and *Hin*dIII restriction sites at the 5′ and 3′ end, respectively, to facilitate re-cloning into the pET28a expression vector, which contains a 6xHis tag. For expression in barley plants we used the construct pUCE_Ubi:HvGBSSIa-GFP:NOS_, which we have described previously (Carciofi *et al.*, 2012). In brief, this construct was made by cloning the original wild-type full length HvGBSSIa cDNA sequence (including the transit peptide) to generate the plasmid pJ241-HvGBSSIa-eGFP (full length HvGBSSIa with a C-terminally linked green fluorescent protein (GFP) tag). Full length HvGBSSIa-eGFP was then recloned into the pUCE plant transformation vector Ubi:USER:NOS (Hebelstrup *et al.*, 2010).

### Site-directed mutagenesis

Mutants for kinetic and *in planta* studies were constructed by using the QuickChange site-directed mutagenesis kit (Agilent Technologies) with pET28a-HvGBSSIa and pJ241-HvGBSSIa-eGFP as templates (respectively for expression either in *E. coli* or in barley), and primers listed in Supplementary Table S1 at *JXB* online. Sequence-confirmed mutants of HvGBSSIa-eGFP were digested with *Pac*I (Fermentas, FastDigest) and recloned into the pUCE vector Ubi:USER:NOS (*Pac*I digested and treated with calf intestine alkaline phosphatase) as described previously (Carciofi *et al.*, 2012).

### Expression and purification of barley starch synthases

The expression vectors of HvGBSSIa or mutants were transformed into *E. coli* Tuner(DE3) cells, which were grown in 1 liter LB medium containing 50 µg ml^–1^ kanamycin to OD ~0.6, cooled on ice for 10 min, induced with 50 µM isopropyl β-D-1-thiogalactopyranoside and incubated overnight at 16 °C. The cell pellet was resuspended in 10 ml buffer A (20 mM Tris–HCl, pH 8.0, 500 mM NaCl, 40 mM imidazole, 1 M UREA, 10% (v/v) glycerol) per gram pellet together with two protease inhibitor tablets (Roche 11 873580001) and two to three drops of antifoam (Sigma-Aldrich). The cell suspension was lysed using a continuous cell disruptor (1.35 kbar, Constant Systems Ltd). DNAseI (2 µg ml^–1^, bovine pancreas, grade II, Roche 101041559001) and MgCl_2_ (final concentration 10 mM) were added to the cell lysate and incubated on ice for 20 min before centrifugation (Beckman, JA-20, 48 400 *g*, 30 min, 4 °C). The filtered supernatant was loaded on a 6 ml HisTrap column (GE Healthcare), equilibrated with buffer A and eluted using a gradient from 0% to 45% buffer B (as buffer A but with 500 mM imidazole). The eluted fractions were mixed with DTT and EDTA, pH 8.0 (final concentration 10 mM of both) and analysed using SDS-PAGE. Fractions containing HvGBSS were pooled, concentrated using a Vivaspin20 centrifugal concentrator (30 kDa molecular mass cut-off) and loaded on a Superdex75 gel-filtration column (GE Healthcare), equilibrated with buffer C (20 mM Tris–HCl, pH 8.0, 150 mM NaCl, 10% (v/v) glycerol, 1 mM DTT, 1 mM EDTA). Fractions were analysed using SDS-PAGE and those containing HvGBSSIa were pooled. The protein concentration was estimated using the Bradford method with bovine immunoglobulin as a reference, divided into 100 µl aliquots, flash-frozen in liquid nitrogen, and stored at –20 °C.

### Coupled glycosyltransferase assay

ADP-glucose, di-ammonium salt was prepared using chemo-enzymatic synthesis as described previously (Cuesta-Seijo *et al.*, 2013). Initial rates were determined by coupling the release of ADP to NADH oxidation via pyruvate kinase and lactate dehydrogenase in a protocol adapted from (Gosselin *et al.*, 1994). Assays were performed in a final volume of 100 µl with the following final concentrations: 50 mM Bicine, pH 8.5, 25 mM potassium acetate, 0.1% (w/v) bovine serum albumin, 2 mM MgCl_2_, 10 mM DTT, 0.375 mM NADH, 0.7 mM phosphorenolpyruvate tricyclohexylammonium salt, 6 U ml^–1^ pyruvate kinase, and 30 U ml^–1^ lactate dehydrogenase (both Sigma-Aldrich, rabbit muscle type II) with 30–800 nM enzyme at 37 °C; 10 mM maltotriose (Sigma-Aldrich M8378) or 1 mg ml^–1^ of glycogen (Sigma-Aldrich G8876, rabbit liver type III) was used as acceptor. Reactions were initiated by addition of 1 mM ADP-glucose. Enzyme concentrations for activity assays were estimated by the method of Bradford with bovine immunoglobulin as a reference. NADH oxidation was monitored by the decrease in absorbance at 340 nm. For single substrate kinetics, the fixed substrate concentration used was >5 times the *K*_m_ value. *k*_cat_ and *K*_m_ values were calculated by fitting the Michaelis–Menten equation to the initial rates (GraphPad Prism version 4.03, GraphPad Software, San Diego, CA, USA).

### Plant transformation

Transgenic callus cultures were induced from immature embryos of *H. vulgare* var. Golden Promise, by *Agrobacterium tumefaciens*-mediated transformation as described previously (Carciofi *et al.*, 2011, 2012). Three plant expression vectors were used: pUCE_Ubi:GBSSIa_^CDC_Alamo^-eGFP for expression of GBSSIa from CDC Alamo, which contains the three amino acid substitutions D219V, M490V, and I491V, was included in this study, to be compared with the wild-type (pUCE_Ubi:GBSSIa_^WT^_-eGFP_) and a vector control (pUCE_Ubi:eGFP_) (Carciofi *et al.*, 2012). The pUCE_Ubi_ plant expression vectors contain the hygromycin phosphotransferase selection marker gene (*hpt*), and the common maize ubiquitin promoter for constitutive expression of the transgene (Hebelstrup *et al.*, 2010). Transgenic barley plants were regenerated from callus in selective medium as described in Carciofi *et al.* (2011). The T_1_ grains from these plants were used for microscopy.

### Western blotting

Developing endosperm was ground in buffer (10 mM Tris–HCl, pH 8.0) in an automatic tissuelyser (FastPrep™ FP120, Thermo Savant) for 20 s. The samples were spun twice at 200 *g* for 2 min. The supernatant was collected, and it was checked with a microscope that it did not contain any starch granules. The pellet, which consisted of starch granules, was washed 10 times with buffer. Protein concentrations were determined with Bradford reagent (Bio-Rad no. 500-0006) using BSA as standard. The samples were mixed with a 6× SDS-loading buffer to reach the following final concentrations: 50 mM Tris–HCl, pH 6.8, 10% glycerol, 1% SDS, 3% β-mercaptoethanol. The samples were boiled for 10 min and the proteins were separated by SDS-PAGE using NuPAGE Novex Bis-Tris Mini Gels (4–12%, Invitrogen) and Bio-Rad Miniprotean II Multiscreen Apparatus according to the manufacturer’s instructions (Bio-Rad bulletin 1721). Proteins were transferred to polyvinylidene fluoride membranes. Rabbit antiserum raised against HvGBSSIa (Carlsberg Laboratory, Copenhagen, Denmark) was used to detect HvGBSSIa at a dilution of (1:1000). Purified antibodies from rabbit serum raised against eGFP (Sigma-Aldrich, G1544) at a dilution of (1:1000) was used to detect eGFP-tagged HvGBSSIa. The secondary antibody (1:5000) was goat anti-rabbit purified IgG coupled with alkaline phosphatase (Sigma-Aldrich, A3687).

### Crossing and genotyping

Transgenic plants with overexpression of the GBSSIa variants were grown in a controlled growth chamber (12–14 °C) under a 16 h day (550 μmol m^–2^ s^–1^)/8 h dark cycle, and with a relative humidity of 90–95%. Spikes with non-mature green anthers were identified and emasculated 2 days before trans-pollination with mature anthers from the three varieties: CDC Alamo, CDC Candle and SB94912 of *H. vulgare*. F_1_ grains were harvested at maturity and a new generation was propagated to obtain mature F_2_ grains. Embryos were removed from the F_2_ grains for genomic DNA purification using the FastDNA^®^ Kit (MP Biomedicals, LLC, OH, USA). The F_2_ grains were genotyped with respect to the GBSSIa gene by PCR as described by (Asare *et al.*, 2012). In brief, the primers Hor_wx_1F (5′-CAAAAAGCGAAGAGGAAGGA-3′) and Hor_wx_1R (5′-AGAATCGAACCAACCGAGTG-3′) were used to identify the GBSSIa genotypes of CDC Candle and SB94912, and the primers Hor_wx_5F (5′-TTTTGCTAGGTGGCCTTCTG-3′) and Hor_wx_5R (5′-TCCGATCACTCAATCATCCA-3′) combined with digestion of the PCR product with *Drd*I were used to identify the CDC Alamo GBSSIa genotype (Asare *et al.*, 2012).

### Epifluorescence and confocal laser scanning microscopy

Endosperm cells and starch granules from T_1_ and F_2_ grains were analysed by fluorescence, Normarski/differential interference contrast (DIC) and transmission light microscopy on a Zeiss Axioplan 2 epifluorescence microscope equipped with an AxioCam MRc5. The filter B1001 (supplied by the manufacturer) was used for detection of eGFP fluorescence and the filter B1000 was used for detection of endosperm cell wall autofluorescence. For transmission light microscopy, starch granules were stained by Lugol’s iodine stain solution (250 mg I_2_, 2.5 g KI, 125 mL ddH_2_O) (Carciofi *et al.*, 2012) to identify the waxy phenotype. Granular localization of GFP-tagged GBSSIa and mutants was also determined using a Leica TCS SP5-X MP UV confocal microscope equipped with filters for eGFP or chlorophyll detection. Endosperm was isolated from developing grains harvested 30 days after pollination and from dry mature grains or mature grains imbibed in sterile ddH_2_O for 2 h. Relative fluorescence from starch granules was determined by using the software supplied by the manufacturer. No fluorescence was detected in starch granules from non-transformed barley grains or in grains from vector control (pUCE_Ubi:eGFP_) transformants.

### Amylose determination and qPCR

The amylose content of barley grains was determined using iodine colorimetry as described previously (Wickramasinghe *et al.*, 2009) and expression levels of *HvGBSSIa* mRNA was determined by qPCR as described previously (Carciofi *et al.*, 2012).

## Results

### Enzymatic activity of HvGBSSIa from CDC Alamo

**Fig. 1.**
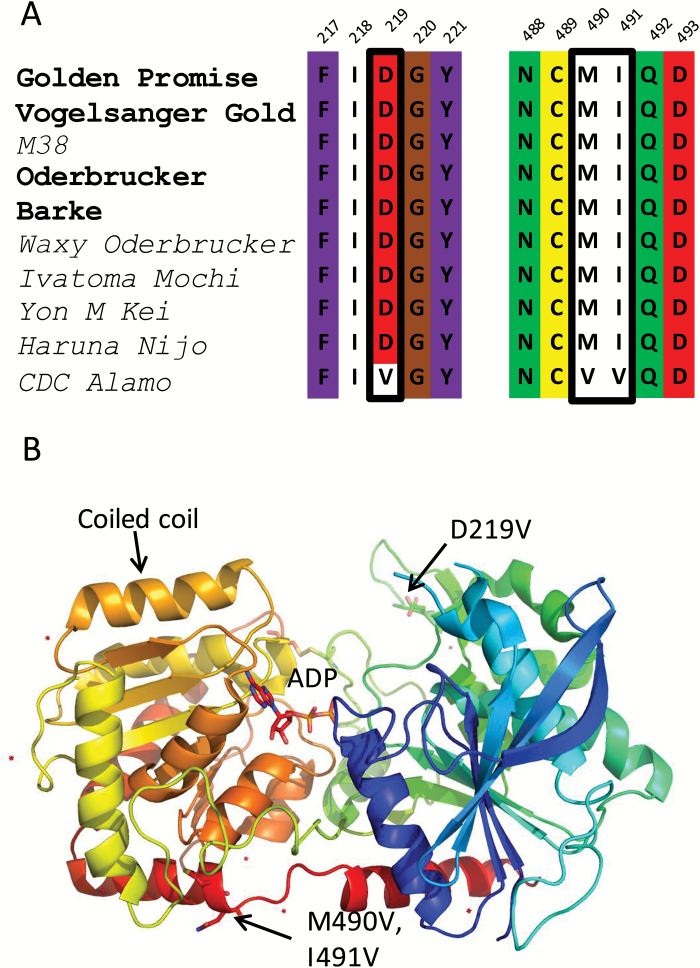
(A) Three unique amino acid substitutions were identified in HvGBSSIa^CDC_Alamo^ (D219V, M490V, and I491V) as compared with a number of other *waxy* and *non-waxy* (in bold) barley varieties. (B) Mapping of these three amino acid substitutions on the structure of the catalytic domain of rice GBSSIa (Momma and Fujimoto, 2012; PDB entry 4VUF). D219V is located on the N-terminal Rossman fold while M490V and I491V are on the C-terminal Rossman fold. None of the three amino acids are positioned in the catalytic site in which ADP is bound, or involved in any other putative substrate, or protein binding sites.

A single SNP which causes an Asp219→Val219 (D219V) mutation in the HvGBSSIa allele of CDC Alamo (HvGBSSIa^CDC_Alamo^) has previously been reported (Asare *et al.*, 2012). However, aligning the cDNA sequence of the HvGBSSIa^CDC_Alamo^ allele with a number of other HvGBSSIa alleles shows that there are also two other SNPs in HvGBSSIa^CDC_Alamo^, which deviate from the consensus sequence of HvGBSSIa (Supplementary Table S2). They cause a Met490→Val490 (M490V) mutation and an Ile491→Val491 (I491V) mutation, respectively (Fig. 1A). The structure of GBSSIa from rice has been determined (Momma and Fujimoto, 2012). We mapped Asp219, M490, and I491 to this structure (Fig. 1B). However, none of these amino acids is located in the presumed catalytic site or in the transit peptide. To test if they are important for the efficiency of GBSSIa starch synthesis, we cloned and successfully expressed six different versions of HvGBSSIa in *Escherichia coli*: (i) a wild-type consensus (HvGBSSIa^WT^) cloned from the *non-waxy* barley line Vogelsanger; (ii) The CDC Alamo variant (HvGBSSIa^CDC_Alamo^) containing all three amino acid substitutions D219V/M490V/I491V; and four different combinations of these, namely (iii) HvGBSSIa^M490V^, (iv) HvGBSSIa ^I491V^, (v) HvGBSSIa ^M490V/I491V^, and (vi) HvGBSSIa ^D219V^. High enzyme yields in *E. coli* (>50 mg l^–1^) were achieved for all six variants. To test the efficiency of each variant, substrate kinetics were determined for all six with either rabbit liver glycogen or ADP-Glc as varying substrate. All five tested variants (HvGBSSIa^D219V^, HvGBSSIa^M490V^, HvGBSSIa^I491V^, HvGBSSIa^M490V/I491V^, and HvGBSSIa^CDC_Alamo^) showed activity *in vitro*, and hence none of the amino acid substitutions resulted in a full loss of catalytic activity (Table 1). The strongest reduction in catalytic activity was observed for HvGBSSIa^CDC_Alamo^, which showed a 90% reduction of *k*_cat_ compared with HvGBSSIa^WT^.

**Table 1.** *Glycosyltransferase activity of various HvGBSSIa variants* in vitro

Enzyme^*c*^	ADP-Glc^*a*^			Glycogen^*b*^		
	*k*_cat_	*K*_m_	*k*_cat_/*K*_m_	*k*_cat_	*K*_m_	*k*_cat_/*K*_m_
	(min^–1^)	(µM)	(min^–1^ µM^–1^)	(min^–1^)	(µg ml^–1^)	(min^–1^ µg^–1^ ml)
HvGBSSIa^WT^	5.8 ± 0.2	160 ± 15	3.65 × 10^–2^ (100%)	6.3 ± 0.2	72 ± 6	8.69 × 10^–2^ (100%)
HvGBSSIa^D219V^	2.76 ± 0.06	127 ± 9	2.17 × 10^–2^ (59%)	3.2 ± 0.2	84 ± 16	3.81 × 10^–2^ (44%)
HvGBSSIa^M490V^	21.2 ± 0.6	71 ± 7	2.99 × 10^–1^ (818%)	19.4 ± 0.8	159 ± 20	1.22 × 10^–1^ (140%)
HvGBSSIa^I491V^	6.7 ± 0.3	114 ± 15	5.88 × 10^–2^ (161%)	6.5 ± 0.3	108 ± 15	6.02 × 10^–2^ (69%)
HvGBSSIa^M490V/I491V^	0.94 ± 0.04	56 ± 8	1.70 × 10^–2^ (47%)	1.3 ± 0.2	175 ± 77	7.43 × 10^–3^ (9%)
HvGBSSIa^CDC Alamo^	0.56 ± 0.03	51 ± 10	1.09 × 10^–2^ (30%)	0.7 ± 0.1	139 ± 62	5.30 × 10^–3^ (6%)

^*a*^ ADP-Glc as the varying substrate; the concentration of rabbit liver glycogen was constant at 1 mg ml^–1^.

^*b*^ Rabbit liver glycogen as the varying substrate; the concentration of ADP-Glc was constant at 1 mM.

^*c*^ Wild-type *k*_cat_ activities correspond to 0.102–0.095 µmol min^–1^ mg^–1^.

### Starch targeting patterns of HvGBSSIa

**Fig. 2.**
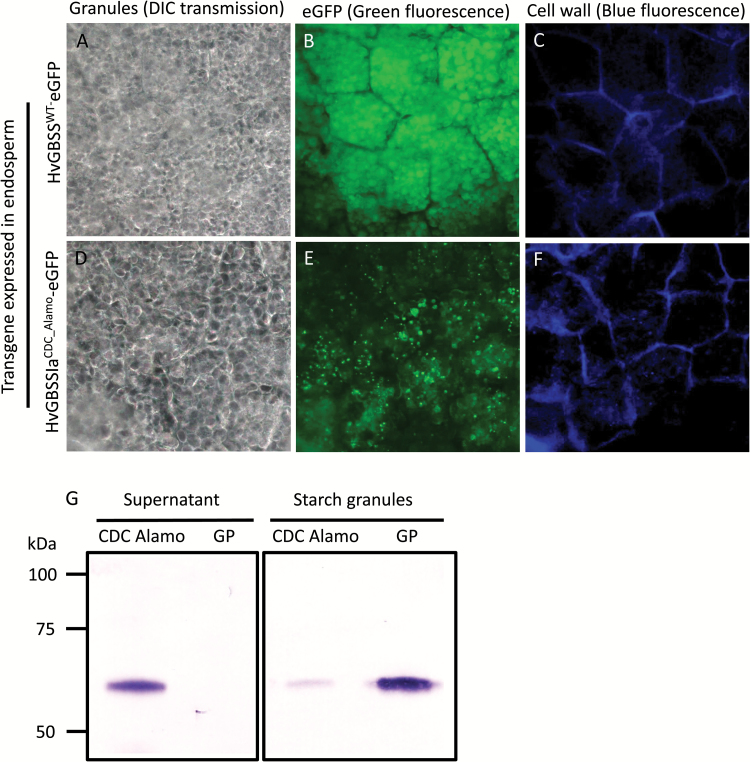
(A–F) Subcellular localization of HvGBSSIa^WT^-eGFP (A–C) and HvGBSSIa^CDC_Alamo^-eGFP (D–F) in 100 µm-thick sections of developing endosperm (30 DAP) of grains from transgenic barley plants. Starch granules were detected with transmitted light using differential interference contrast (DIC) settings (A, D); HvGBSSIa^CDC_Alamo^-eGFP and HvGBSSIa^WT^-eGFP were detected as green epifluorescence (B, E); cell walls were detected as blue epifluorescence (C, F). HvGBSSIa^WT^-eGFP is strictly targeted to starch granules (B), whereas HvGBSSIa^CDC_Alamo^-eGFP is mostly targeted into unknown subcellular bodies (E). (G) Western blotting with GBSSI antiserum confirms that HvGBSSIa is strictly targeted to starch granules in the endosperm of Golden Promise, whereas it is mostly found in the supernatant of the endosperm cell fraction of CDC Alamo. All lanes are from the same blot.

**Fig. 3.**
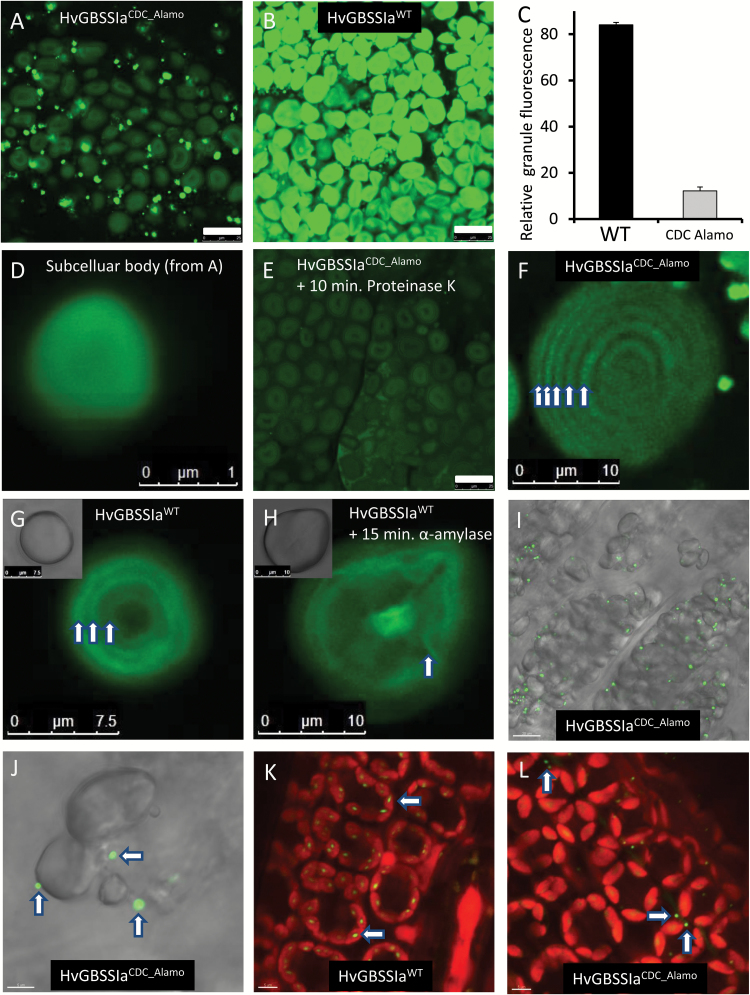
Confocal laser scanning microscopy of starch granules from the endosperm of developing grains at the late grain filling stage (30 DAP) of transgenic barley plants expressing HvGBSSIa^WT^-eGFP (B, G, H, K) or HvGBSSIa^CDC_Alamo^-eGFP (A, E, F). (A), (B), (E) and (F) are recorded with same sensitivity, whereas (D), (G) and (H) were recorded with lower sensitivity to avoid oversaturation. (C) The relative fluorescence intensities were much stronger in HvGBSSIa^WT^-eGFP starch granules than in HvGBSSIa^CDC_Alamo^-eGFP starch granules (*n*=10). Bars indicate standard errors. (D) Magnified subcellular body from HvGBSSIa^CDC_Alamo^-eGFP endosperm cells from panel A. (E) The subcellular bodies in panel (A) disappeared after treatment with proteinase K (1 mg ml^–1^ in H_2_O) for 10 min. (G, H) Both HvGBSSIa^WT^-eGFP and HvGBSSIa^CDC_Alamo^-eGFP are localized into concentric spheres (indicates by arrows) within starch granules. (H) These concentric spheres became blurred (indicated by the arrow) upon treatment with α-amylase. (I, J) Transmission light was used to visualize subcellular structures in thin sections of endosperm cells simultaneously with localization of HvGBSSIa^CDC_Alamo^-eGFP recorded as overlaid eGFP fluorescence. (K, L) HvGBSSIa^WT^-eGFP (green; K) was located in granules inside chloroplasts (red fluorescence) in leaves whereas HvGBSSIa^CDC_Alamo^-eGFP (L) was mostly localized in subcellular bodies (green, indicated by arrows) outside of the red chloroplasts. The scale bars in (A, B, E) are 25 µm; the scale bar in (I) is 20 µm; the scale bars in (F, H) are 10 µm; the scale bar in (G) is 7.5 µm; the scale bars in (J, K, L) are 5 µm; and the scale bar in (D) is 1 µm.

**Fig. 4.**
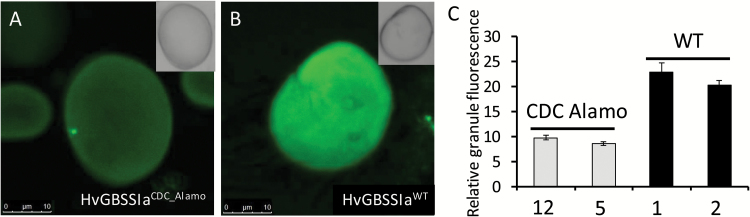
(A, B) Confocal laser scanning microscopy of starch granules from mature dry grains of transgenic barley plants expressing HvGBSSIa^CDC_Alamo^-eGFP (A) and HvGBSSIa^WT^-eGFP (B). (C) Relative fluorescence intensities in HvGBSSIa^WT^-eGFP and HvGBSSIa^CDC_Alamo^-eGFP starch granules (*n*=10) from two different lines each: HvGBSSIa^CDC_Alamo^-eGFP (lines 12 and 5) and HvGBSSIa^WT^-eGFP (lines 1 and 2). Bars indicate standard errors.

HvGBSSIa^CDC_Alamo^ had a strong reduction in catalytic activity, but was not fully inactive. To examine the subcellular localization patterns of GBSSIa, we generated transgenic barley plants (var: Golden Promise, GP) from calli transformed with constructs for overexpression of either HvGBSSIa^CDC_Alamo^ or HvGBSSIa^WT^ as fusion proteins tagged with eGFP. GBSS-type enzymes are strictly associated with starch granules. In our previous work, we found that HvGBSSIa^WT^-eGFP fusion protein expressed in callus cells was strictly localized to starch granules (Carciofi *et al.*, 2012), whereas such visual localization of GBSS within storage starch granules from tubers or grains has not previously been reported. Barley grain starch has a bimodal granule morphology consisting of large disc-shaped granules of 15–50 µm (A-type) and small granules of 2–3 µm (B-type) (Jane *et al.*, 1994; Carciofi *et al.*, 2012). Barley callus cell starch granules are much smaller (<3 µm) than the large A-type granules from grains (Carciofi *et al.*, 2012). We therefore studied the cellular localization pattern of HvGBSSIa^CDC_Alamo^-eGFP and HvGBSSIa^WT^-eGFP in endosperm cells in grains from two independent transgenic barley lines for each of the HvGBSSIa varieties. Starch granules were detected with transmitted light using DIC settings. HvGBSSIa^CDC_Alamo^-eGFP and HvGBSSIa^WT^-eGFP were detected as green epifluorescence. Cell walls were detected as blue fluorescence. In endosperm cells of developing grains (30 days after pollination (DAP)), HvGBSSIa^WT^-eGFP was strictly associated with the starch granules (Fig. 2A–C), whereas HvGBSSIa^CDC_Alamo^-eGFP showed a deficient starch targeting pattern with localization into smaller subcellular bodies rather than into starch granules (Fig. 2D–F). Western blotting with antiserum against HvGBSSI on either the protein fraction of the supernatant or on starch granules purified from developing endosperm showed bands with similar size in the supernatant and starch granules from CDC Candle or starch granules of GP. These bands correspond well to the expected size (59 kDa) of HvGBSSIa without a transit peptide. While GBSSIa was strictly associated with starch granules in GP, GBSSIa in CDC Alamo was detected mainly in the supernatant, with only a smaller quantity in the starch granules (Fig. 2G). Western blotting with eGFP antibody similarly confirmed that transgenic HvGBSSIa^WT^-eGFP was located in purified starch granules, whereas HvGBSSIa^CDC_Alamo^-eGFP was mostly associated with the supernatant, rather than starch granules (Supplementary Fig. S1). The localizations, as detected by the eGFP signal of HvGBSSIa^WT^-eGFP and HvGBSSIa^CDC_Alamo^-eGFP into starch granules and the smaller subcellular bodies, were further investigated by confocal laser scanning microscopy (Fig. 3). This showed that for both HvGBSSIa^WT^-eGFP and HvGBSSIa^CDC_Alamo^-eGFP fluorescence was detected in both large (A-type) and small (B-type) starch granules. However, HvGBSSIa^WT^-eGFP (Fig. 3A) was more strongly associated with starch granules than HvGBSSIa^CDC_Alamo^-eGFP (Fig. 3B) when comparing relative difference in granule fluorescence (Fig. 3C). The subcellular bodies associated with HvGBSSIa^CDC_Alamo^-eGFP were mostly disc shaped with less fluorescence in the center and a diameter of 0.5–3 µm (Fig. 3D). They disappeared completely after a 10 min treatment with proteinase K, leaving both large (A-type) and small (B-type) starch granules intact (Fig. 3E). This suggests that they may be protein bodies. Interestingly, both HvGBSSIa-eGFP types were not uniformly distributed within the starch granules but were concentrated in internal concentric spheres (Fig. 3F–G). These spheres became blurred after a 15 min treatment with α-amylase (Fig. 3H; only HvGBSSIa^WT^ is shown, although HvGBSSIa^CDC_Alamo^ showed a similar blurring pattern). We recorded both transmitted light (with DIC) and eGFP to simultaneously visualize endosperm subcellular structures and HvGBSSIa^CDC_Alamo^-eGFP subcellular bodies in thin (50 µm) sections of developing endosperm (Fig. 3I, J). While some of the subcellular bodies seemed to be located away from starch granules (Fig. 3J), we were unable to determine if the subcellular bodies were located inside or outside of the amyloplast membrane. However, in leaves where HvGBSSIa^CDC_Alamo^-eGFP and HvGBSSIa^WT^-eGFP were also expressed, we observed that HvGBSSIa^WT^-eGFP was localized inside chloroplasts (Fig. 3K), whereas HvGBSSIa^CDC_Alamo^-eGFP was mostly located outside of chloroplasts (Fig. 3L). In dry, post-harvest mature grains there was only a slight tendency for distribution into concentric spheres in both HvGBSSIa^WT^-eGFP and HvGBSSIa^CDC_Alamo^-eGFP starch granules (Fig. 4A, B) and the difference in relative levels of fluorescence between HvGBSSIa^CDC_Alamo^-eGFP and HvGBSSIa^WT^-eGFP starch granules was smaller (Fig. 4C) than during endosperm development (Fig. 3C).

### Deficient starch targeting of HvGBSSIa^CDC_Alamo^-eGFP is associated with the waxy phenotype

**Fig. 5.**
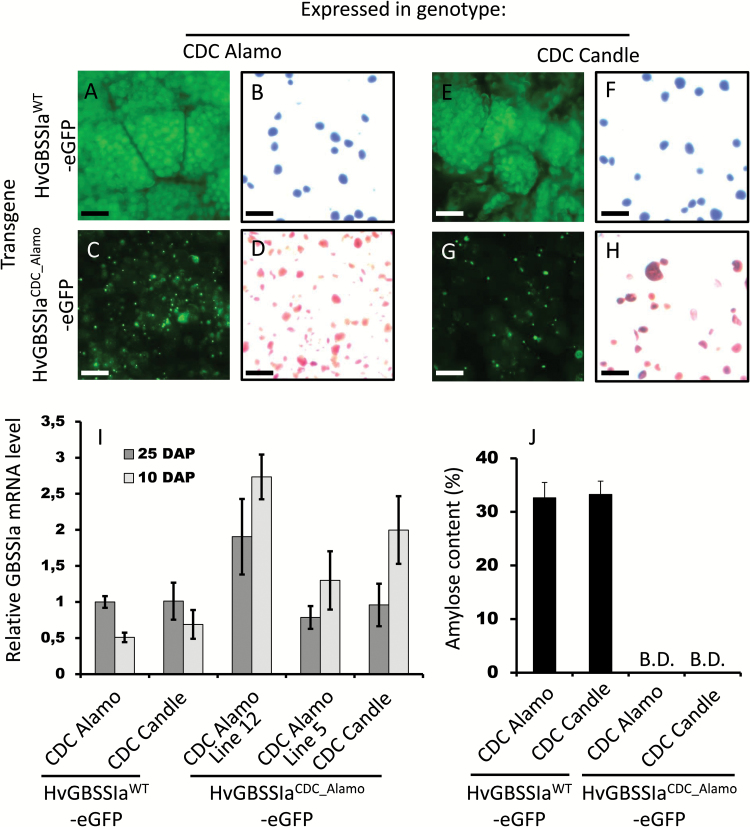
(A–H) eGFP fluorescence from endosperm cells (A, C, E, G) and iodine-stained starch granules (B, D, F, H) from developing grains (30 DAP) of plants with transgenic expression of HvGBSSIa^WT^-eGFP (A, B, E, F) or HvGBSSIa^CDC_Alamo^-eGFP (C, D, G, H) in the three *waxy* cultivars CDC Alamo (A–D), CDC Candle (E–H) and SB94912. The results for SB94912 were identical to those of CDC Candle, so for simplicity, only CDC Candle is shown. The scale bars in (A–H) are 50 µm. (I) Relative gene expression levels of *HvGBSSIa* determined by qPCR in developing grains (10 and 25 DAP) from plants with overexpression of either HvGBSSIa^WT^-eGFP or HvGBSSIa^CDC_Alamo^-eGFP (two different lines: 12 and 5) in the cultivars CDC Alamo and CDC Candle. (J) Amylose content in starch isolated from dry mature grains of plants expressing either HvGBSSIa^WT^-eGFP or HvGBSSIa^CDC_Alamo^-eGFP in CDC Alamo or CDC Candle.

Golden Promise is a non-waxy variety with fairly high amylose content, and overexpression of either HvGBSSIa^WT^-eGFP or HvGBSSIa^CDC_Alamo^-eGFP in Golden Promise did not change the amylose content, suggesting that GBSSIa enzyme activity may already be saturated in this variety (not shown). Therefore, to further demonstrate the link between the deficient GBSSIa^CDC_Alamo^-eGFP starch targeting pattern with the waxy phenotype of CDC Alamo, we crossed the transgenic barley plants expressing either GBSSIa^CDC_Alamo^-e GFP or HvGBSSIa^WT^-eGFP with either CDC Alamo or either of the *near-waxy* barley varieties CDC Candle or SB94912 (♀ Golden Promise_Ubi:GBSSIa^(WT *or* CDC_Alamo)^-eGFP × ♂ CDC Alamo *or* CDC Candle *or* SB94912). Both of the HvGBSSIa^CDC_Candle^ and HvGBSSIa^SB94912^ alleles contain a deletion in the promoter and have low expression levels of HvGBSSIa enzyme imparting a near-waxy starch phenotype (Asare *et al.*, 2012). The near-waxy starch phenotype can be identified by a screen with iodine staining of endosperm flour, which will reveal *near-waxy* starch granules staining with a red color, in contrast to *non-waxy* starch granules, which are blue. Parents and F_2_ grains of the crosses were genotyped with respect to the endogenous *HvGBSSIa* gene as described in ‘Materials and methods’ to identify F_2_ grains that are homozygous for one of the endogenous *waxy* alleles *HvGBSSIa*^*CDC_Alamo*^, *HvGBSSIa*^*CDC_Candle*^ or *HvGBSSIa*^*SB94912*^. Expression of HvGBSSIa^CDC_Alamo^-eGFP and HvGBSSIa^WT^-eGFP in the endosperm cells of F_1_ and segregating F_2_ grains was detectable with fluorescence microscopy as described above for T_1_ grains, and also showed localization patterns identical to those observed in T_1_ grains: HvGBSSIa^WT^-eGFP was strictly associated with starch granules (Fig. 5A, E), whereas HvGBSSIa^CDC_Alamo^-eGFP showed a deficient starch targeting pattern with localization into subcellular bodies rather than into starch granules (Fig. 5C, G). Furthermore, there was a difference between HvGBSSIa^CDC_Alamo^-eGFP and HvGBSSIa^WT^-eGFP in their ability to rescue the waxy phenotype of CDC Alamo, CDC Candle or SB94912: transgenic expression of HvGBSSIa^WT^-eGFP rescued the three *waxy* varieties by making *non-waxy* blue-staining granules (Fig. 5B, F) with an amylose content above 30% in mature grains (Fig. 5J), whereas the three *waxy* lines with over-expression of HvGBSSIa^CDC_Alamo^-eGFP retained red-staining *waxy* starch granules with an amylose content below detection level (Fig. 5J). The effect was the same in the three different cultivars, showing that the deficient starch targeting pattern of HvGBSSIa^CDC_Alamo^-eGFP as well as the inability of HvGBSSIa^CDC_Alamo^-eGFP to rescue the waxy phenotype is independent of both the endogenous *HvGBSSIa* locus and the genetic background in the three different *waxy* varieties used in this study. The results for SB94912 were identical to those of CDC Candle. So for simplicity, only CDC Candle is shown in Fig. 5. To ensure that the observed deficiencies of HvGBSSIa^CDC_Alamo^-eGFP was not due to a difference in expression efficiency between the *HvGBSSIa*^*CDC_Alamo*^*-eGFP* and *HvGBSSIa*^*WT*^*-eGFP* transgene, mRNA expression levels of total *HvGBSSIa* were measured by qPCR, which demonstrated that expression of *HvGBSSIa*^*CDC_Alamo*^*-eGFP* was not significantly lower than expression of *HvGBSSIa*^*WT*^*-eGFP*. Two independent lines of HvGBSSIa^CDC_Alamo^-eGFP expression were used (lines 5 and 12) and both failed to complement *waxy* in any of the three cultivars.

## Discussion

The amylose-free phenotype of barley cv. CDC Alamo has been presumed to be the result of a complete elimination of HvGBSSIa activity caused by a single SNP alteration from adenine to thymine, which replaces the aspartic acid with the hydrophobic amino acid valine at position 219 (Patron *et al.*, 2002; Asare *et al.*, 2012). This mutation was never tested *in vitro* and only measurement of starch synthase activity associated with granules in developing endosperm of barley cv. CDC Alamo suggested that most or all of GBSSIa protein was inactive (Patron *et al.*, 2002). In addition, a re-examination of the gene entry of barley cv. CDC Alamo (AF486519) revealed two extra SNPs that also resulted in two mutations, M490V and I491V (Fig. 1A).

Examination of Asp219, Met490, and Ile491 in a modeled structure based on the published structure of rice GBSSIa, which shares very close homology with barley HvGBSSIa (Momma and Fujimoto, 2012), did not provide an immediate explanation as to why HvGBSS in CDC Alamo should be inactive (Fig. 1B). GBSSIa belongs to the GT5 family of glycosyltransferases in the CAZy database (Coutinho *et al.*, 2003), which adopts the characteristic GT-B fold (double Rossmann fold). The interface between the two Rossmann folds forms the catalytic site wherein an ADP molecule was identified in the rice GBSSIa structure (Momma and Fujimoto, 2012). Asp219 is neither positioned in this catalytic site nor involved in any putative substrate binding, but is located in the 380s loop of starch synthases; a region that is highly variable among all starch synthase gene classes (Leterrier *et al.*, 2008). However, Asp219 itself is conserved among different plant GBSS genes (Leterrier *et al.*, 2008), indicating that it may have some importance for protein structure and function. Asp219 (HvGBSS numbering) is also in close proximity with the basic Arg196 and Lys229 residues, which could potentially be disturbed by the Asp219→Val substitution in HvGBSSaI^CDC_Alamo^. Met490 and Ile491 are both located on the second to last α-helix in the structure (the last one of the C-terminal Rossman fold). Met490 is buried in the structure of rice GBSSIa where Ile491 (Asn in Rice GBSS) is surface exposed (Momma and Fujimoto, 2012). Met490 is conserved among cereal starch synthase amino acid sequences from all five starch synthase gene classes (Leterrier *et al.*, 2008).

The three identified substitutions D219V, M490V, and I491V were selected as targets for a mutational study of HvGBSSIa to explain the amylose-free phenotype of CDC Alamo. The specific activity of HvGBSSIa^WT^ correlated well with previous studies of purified maize GBSS (Macdonald and Preiss, 1983, 1985). HvGBSSIa^D219V^ showed only a small reduction in activity (44–59%) compared with wild-type (HvGBSSIa^WT^), while HvGBSSIa^M490V/I491V^ (9–47%) and HvGBSSIa^CDC_Alamo^ (6–30%) activities were much lower. The catalytic properties of HvGBSSIa^I491V^ were essentially unchanged compared with wild-type; however, HvGBSSIa^M490V^ differed significantly from the wild-type by having at least three-fold higher *k*_cat_. The *K*_m_ value towards ADP-glucose HvGBSSIa was in the same range as those previously determined for maize GBSS (Macdonald and Preiss, 1985), but notably lower than that of potato (Edwards *et al.*, 1999) and *Chlamydomonas reinhardtii* GBSS (Delrue *et al.*, 1992) (10- and 23-fold lower, respectively). In summary, the combination of the three SNPs in the *HvGBSSIa* gene of CDC Alamo seems to lead to strong reduction in catalytic activity of the enzyme.

Our results indicate that the waxy phenotype is also associated with deficient targeting of HvGBSSIa into starch granules in CDC Alamo. We used eGFP tagging to demonstrate that a consensus HvGBSSIa from non-waxy cultivars is targeted to starch granules, where it is localized into concentric spheres. This is well in line with the general consensus that GBSS-type enzymes are bound tightly to starch granules within the internal granular matrix (Tatge *et al.*, 1999; Wang *et al.*, 2013). Concentric spheres of similar morphology were observed in wheat, maize, and potato starch in a confocal laser scanning microscopy study of starch treated with a protein-specific dye (3-(4-carboxybenzoyl) quinoline-2-carboxaldehyde) (Han and Hamaker, 2002). In line with our observations, these spheres are likely to be GBSSIa, as the concentric spheres were not observed in waxy maize and amylose-free potato starch, which correlated with the lack of expression of GBSSIa (Han and Hamaker, 2002). In contrast, the targeting of HvGBSSIa is deficient in CDC Alamo. Western blotting with HvGBSSIa antiserum showed that HvGBSSIa^CDC_Alamo^ fails to be fully targeted to starch granules in CDC Alamo, where a major fraction of HvGBSSIa^CDC_Alamo^ is found in the soluble fraction of developing endosperms (Fig. 2G). Transgenic expression of eGFP-tagged HvGBSSIa^WT^ or HvGBSSIa^CDC_Alamo^ confirmed that HvGBSSIa^CDC_Alamo^ is unable to target correctly to starch granules and to synthesize amylose *in vivo* (Fig. 5). HvGBSSIa is generally targeted to amyloplasts by a transit peptide that is cleaved when the protein is transported through the amyloplast membrane. Our previous work has demonstrated that a transit peptide, with an identical 69 amino-acid sequence to that of HvGBSSIa^WT^ and HvGBSSIa^CDC_Alamo^ (which have identitical gene sequences for the transit peptide), effectively transports transgenic eGFP to the amyloplast of developing barley endosperm cells (Hebelstrup *et al.*, 2010). This suggests that the incorrect targeting of HvGBSSIa^CDC_Alamo^ is not due to a deficient transit peptide. This is supported by the western blot (Fig. 2G), which shows that the soluble fraction of HvGBSSIa^CDC_Alamo^ in CDC Alamo has an identical size to that of the HvGBSSIa^CDC_Alamo^ that is correctly targeted to starch granules. This implies that the transit peptide must have been correctly removed from the soluble fraction of HvGBSSIa^CDC_Alamo^.

GBSSI seems to be the predominant protein in storage starch granules (Wang *et al.*, 2013). In contrast, soluble starch synthases and starch branching enzymes form complexes (Tetlow *et al.*, 2008; Liu *et al.*, 2012). However, GBSS-type enzymes do not contain any identified starch binding modules, nor do they interact in the mentioned complexes with the soluble starch synthases or starch branching enzymes containing starch binding modules. In Arabidopsis leaves, amylose synthesis is dependent on a GBSS type that is targeted to transient starch granules by interaction with the protein PTST (protein targeting to starch), which contains a carbohydrate binding module, and loss-of-function *ptst* mutant Arabidopsis plants synthesize a *waxy*-like type of transient starch in their leaves (Seung *et al.*, 2015). A PTST gene homolog exists in the barley genome, and therefore the same mechanism may be necessary for correct targeting of HvGBSSIa to storage starch granules and synthesis of amylose in barley grain endosperm. This also demonstrates that mutations in other genes than GBSS can contribute to the waxy phenotype. However, in CDC Alamo, the three identified mutations in HvGBSSIa seem to be the single cause of deficient targeting to starch granules and the waxy phenotype, because HvGBSSIa^CDC_Alamo^ was also incorrectly targeted and unable to rescue the waxy phenotype when expressed in the cultivars CDC Candle and SB94912 (Fig. 5), which are *waxy* due to a deletion in the gene promoter and subsequently lack HvGBSSIa enzyme (Asare *et al.*, 2012). Similarly, the waxy phenotype could be rescued in CDC Alamo by expression of functional HvGBSSIa^WT^ resulting in >30% amylose content. Given that binding of GBSS with PTST-type proteins is necessary for correct targeting of GBSS and synthesis of amylose (Seung *et al.*, 2015), it is possible that one or more of the three amino acid substitution (D219V, M490V, and I491V) in HvGBSSIa^CDC_Alamo^ in barley prevents binding to a putative PTST protein, whereby HvGBSSIa^CDC_Alamo^ is incorrectly targeted. However, the binding of GBSS to PTST in Arabidopsis was mediated through a coiled-coil domain identified on the distal helix (Fig. 5B), which is not in the structural area of the three amino acid substitutions of HvGBSSIa^CDC_Alamo^.

We cannot exclude the possibility that the deficient targeting of HvGBSSIa in CDC Alamo is caused indirectly by the partial loss of catalytic activity. It can also be speculated that HvGBSSIa is a less processive variant, which causes it to fail to remain bound to (or within) the starch granules after correct targeting. The cellular structure of the subcellular bodies where HvGBSSIa^CDC_Alamo^-eGFP is located is not clear (Figs 2, 3 and 5). Their sensitivity to proteinase K indicates that they are protein-rich bodies. In leaves, at least some of them are located outside of chloroplasts (Fig. 3L). In endosperm cells, it was not clear if they are located outside or on the inside of the amyloplast membrane. However, as discussed above, western blotting suggests that their transit peptide has been correctly removed, indicating that they have been translocated across a plastid membrane. It cannot be excluded that they represent more than one type of cellular structure. They do not seem to be breakdown products of HvGBSSIa, since the western blot showed a single intact protein in the soluble fraction of CDC Alamo with a size similar to that of the HvGBSSIa in the starch granules in Golden Promise (Fig. 2G). Similar results were obtained for a transgene system where HvGBSSIa^CDC_Alamo^-eGFP expressed in endosperm located mostly to the supernatant with a similar size to that of the correctly targeted HvGBSSIa^WT^-eGFP and neither showed signs of breakdown products (Supplementary Fig. S1). In summary, our work suggests that the *waxy* phenotype of CDC Alamo is associated with a combination of a strong reduction of catalytic activity of HvGBSSIa, as well as a deficiency of either targeting or retaining HvGBSSIa within the matrix of starch granules.

## Supplementary Material

Supplementary Data

## Abbreviations:

DIC: differential interference contrast
eGFP: enhanced green fluorescent protein
GBSS: granule-bound starch synthase
GP: Golden Promise
SNP: single nucleotide polymorphism
SS: starch synthase.
