# Supplementary material for: Waxy and non-waxy barley cultivars exhibit differences in the targeting and catalytic activity of GBSS1a
Source: J Exp Bot. 2017 Feb 11;68(5):931–41. doi: 10.1093/jxb/erw503 (PMC5441850; doi:10.1093/jxb/erw503)
Supplement: Supplementary Data [file erw503_Supplementary_Data.zip › supplementary_figure_S1_tables_S1_S2.pdf]

Supplementary Fig. S1

Golden promise barley plants were transformed to express either HvGBSSIa$^{CDC\ Alamo}$-eGFP or HvGBSSIa^{WT}-eGFP. The localiztion of the transgene (expected size 86.2 kD) in either the supernatant or the starch granules from deverloping endosperm cells was determined with an eGFP antibody.

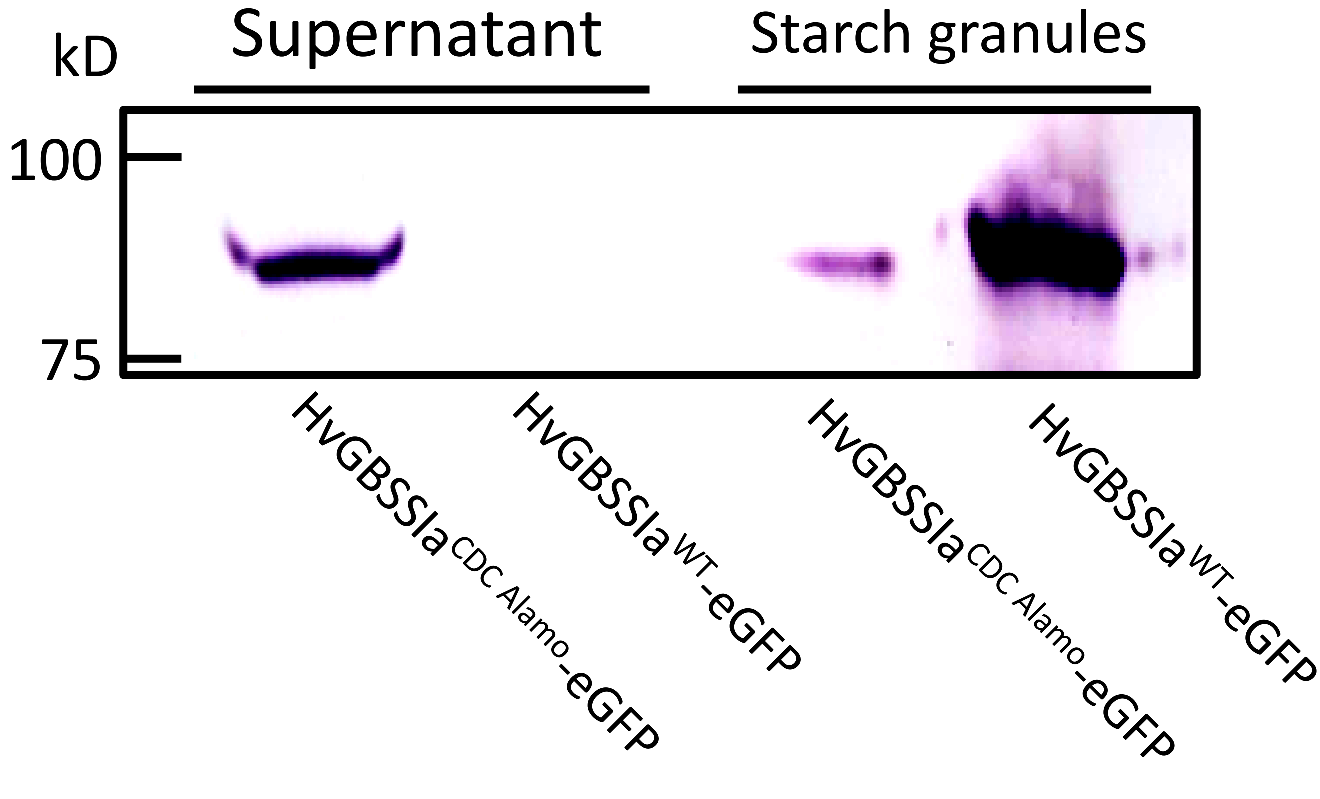

Supplementary Table S1

pET28a HvGBSSIa	
D219V	ctcgtttgacttcatcgttggctatgacaaaccag
D219_antisense	ctggtttgtcatagccaacgatgaagtcaaacgag
M490V	ggaaatggtgaagaattgcgtgattcaggacctgtcatg
M490V_antisense	catgacaggtcctgaatcacgcaattcttcaccatttcc
I491V	tggtgaagaattgcatggttcaggacctgtcatgg
I491V_antisense	ccatgacaggtcctgaaccatgcaattcttcacca
M490VI491V	ggaaatggtgaagaattgcgtggttcaggacctgtcatggaa
M490VI491V_antisense	ttccatgacaggtcctgaaccacgcaattcttcaccatttcc
pJ241 HvGBSSIa-eGFP	
D219V	tccttcgacttcattgtcggctacgacaagcc
D219V_antisense	ggcttgtcgtagccgacaatgaagtcgaagga
M490V	agatggtcaagaactgcgtgatccaggatctctcc
M490V_antisense	ggagagatcctggatcacgcagttcttgaccatct
I491V	ggtcaagaactgcatggtccaggatctctcctg
I491V_antisense	caggagagatcctggaccatgcagttcttgacc
M490V/I491V	agatggtcaagaactgcgtggtccaggatctctcctgg
M490V/I491V_antisense	ccaggagagatcctggaccacgcagttcttgaccatct

Supplementary Table S2

```
Vogelsanger gold TCGTCCTTCGACTTCATTGACGGCTACGACAAGCCCGTGGAGGGGCGCAAGATCAACTGG        900
                  S  S  F  D  F  I  D  G  Y  D  K  P  V  E  G  R  K  I  N  W
M38              TCGTCCTTCGACTTCATTGACGGCTACGACAAGCCCGTGGAGGGGCGCAAGATCAACTGG
Oderbruker       TCGTCCTTCGACTTCATTGACGGCTACGACAAGCCCGTGGAGGGGCGCAAGATCAACTGG
Barke            TCGTCCTTCGACTTCATTGACGGCTACGACAAGCCCGTGGAGGGGCGCAAGATCAACTGG
SB 85750         TCGTCCTTCGACTTCATTGACGGCTACGACAAGCCCGTGGAGGGGCGCAAGATCAACTGG
Waxy Oderbrucker TCGTCCTTCGACTTCATTGACGGCTACGACAAGCCCGTGGAGGGGCGCAAGATCAACTGG
Iyatoma_Mochi    TCGTCCTTCGACTTCATTGACGGCTACGACAAGCCCGTGGAGGGGCGCAAGATCAACTGG
Yon M Kei        TCGTCCTTCGACTTCATTGACGGCTACGACAAGCCCGTGGAGGGGCGCAAGATCAACTGG
Haruna Nijo      TCGTCCTTCGACTTCATTGACGGCTACGACAAGCCCGTGGAGGGGCGCAAGATCAACTGG
CDC alamo        TCGTCCTTCGACTTCATTGTCGGCTACGACAAGCCCGTGGAGGGGCGCAAGATCAACTGG
                  S  S  F  D  F  I  V  G  Y  D  K  P  V  E  G  R  K  I  N  W
Morex(EST)       TCGTGCTTCGACTTCATTGACGGCTACTACAAGCCCGCGGAGGGGCGCAAGATCAACTGG
                  S  C  F  D  F  I  D  G  Y  Y  K  P  A  E  G  R  K  I  N  W
                 **** **************:******* ********* *********************
```

```
Golden Promise   GTCGGCACGCCGGCGTACCAGGAGATGGTCAAGAACTGCATGATCCAGGATCTCTCCTGG        1692
                  V  G  T  P  A  Y  Q  E  M  V  K  N  C  M  I  Q  D  L  S  W
Vogelsanger gold GTCGGCACGCCGGCGTACCAGGAGATGGTCAAGAACTGCATGATCCAGGATCTCTCCTGG
M38              GTCGGCACGCCGGCGTACCAGGAGATGGTCAAGAACTGCATGATCCAGGATCTCTCCTGG
Oderbruker       GTCGGCACGCCGGCGTACCAGGAGATGGTCAAGAACTGCATGATCCAGGATCTCTCCTGG
Barke            GTCGGCACGCCGGCGTACCAGGAGATGGTCAAGAACTGCATGATCCAGGATCTCTCCTGG
SB 85750         GTCGGCACGCCGGCGTACCAGGAGATGGTCAAGAACTGCATGATCCAGGATCTCTCCTGG
Waxy Oderbrucker GTCGGCACGCCGGCGTACCAGGAGATGGTCAAGAACTGCATGATCCAGGATCTCTCCTGG
Iyatoma Mochi    GTCGGCACGCCGGCGTACCAGGAGATGGTCAAGAACTGCATGATCCAGGATCTCTCCTGG
Yon M Kei        GTCGGCACGCCGGCGTACCAGGAGATGGTCAAGAACTGCATGATCCAGGATCTCTCCTGG
Haruna Nijo      GTCGGCACGCCGGCGTACCAGGAGATGGTCAAGAACTGCATGATCCAGGATCTTTCCTGG
                  V  G  T  P  A  Y  Q  E  M  V  K  N  C  M  I  Q  D  L  S  W
CDC alamo        GTCGGCACGCCGGCGTACCAGGAGATGGTCAAGAACTGCGTGGTCCAGGATCTCTCCTGG
                  V  G  T  P  A  Y  Q  E  M  V  K  N  C  V  V  Q  D  L  S  W
Optic(EST)       GTCGGCACGCCGGCGTACCAGGAGATGGTCAAGAACTGCACGATCCACGATCTCTCC---
                  V  G  T  P  A  Y  Q  E  M  V  K  N  C  T  I  H  D  L  S
                 ***************************************. *.**** ***** ***
```

Alignment of selected barley GBSSI sequences from various normal and waxy cultivars. Two sequence regions are presented covering the three SNPs of barley cv. CDC Alamo. The *waxy / near-waxy* cultivars (M38 (X676772), SB 85750 (AF486518), Waxy Oderbrucker (AF486515), Haruna Nijo (AK366209), Iyatoma Mochi (AF486517), Yon M Kei (AF486516), CDC Alamo (AF486519)) are highlighted in bold. The *non-waxy* cultivars are Vogelsanger gold (X07932), Barke (FN179380), Oderbrucker (AF486514), Golden Promise (KC963114), Optic (BQ759028), and Morex (BG299297).
